# Examination of the lifestyle of head injury patients using the Frenchay Activities Index

**DOI:** 10.20407/fmj.2022-036

**Published:** 2023-08-28

**Authors:** Masayuki Yamada, Toshio Teranishi, Megumi Suzuki, Kei Ohtsuka, Mihoko Ito, Akiko Maeda, Yoshikiyo Kanada

**Affiliations:** Faculty of Rehabilitation, School of Health Sciences, Fujita Health University, Toyoake, Aichi, Japan

**Keywords:** TBI, FAI, APDL

## Abstract

**Objectives:**

The purpose of this study was to capture the lifestyle characteristics of traumatic brain injury (TBI) patients by administering the Frenchay Activities Index (FAI), a self-assessment questionnaire used for assessing life-related activities, among TBI patients.

**Methods:**

This study was conducted using the revised FAI Self-Assessment Form, administering an interview-based questionnaire survey to TBI patients and healthy participants. The target group comprised 60 traumatic brain injury patients who had progressed from at least 1 year since the injury, with a comparison group of 788 healthy participants.

**Results:**

A comparison of FAI scores between TBI patients and healthy participants by questionnaire revealed that TBI patients exhibited significantly higher FAI scores than healthy participants for outdoor walking and transportation use (Mann–Whitney U test, *P*<0.01). A comparison by occupation revealed that TBI patients were significantly less among the unemployed and other categories (Mann–Whitney U test, *P*<0.01). For office workers, government employees, high school students, and college students, scores were higher among TBI patients compared with healthy participants, although the differences were not significant.

**Conclusions:**

Although TBI patients were less active than healthy participants in life-related activities, their scores for cleaning up after meals and cleaning and putting things in order were close to those of healthy participants, indicating that TBI patients cannot be considered to be inactive. The findings also revealed differences in TBI patients’ engagement in life-related activities and frequency of activities depending on their social position.

## Introduction

Patients with traumatic brain injury (TBI) often have difficulty attending school, performing household chores, and finding employment because of the emergence of personality changes and emotional disturbances after brain injury, as well as difficulties acquiring new knowledge and skills.^1^ The majority of TBI patients are between the ages of 15 and 24 years, and the question of how to live a long life after a brain injury and maintain participation in society is a major concern for TBI patients and their families. Understanding post-discharge behavior related to activities of daily living (ADL) among TBI patients who have reintegrated into the community may be helpful for therapists considering treatment goals and objectives.

The functional independence measure (FIM)^2–4^ and Barthel Index^5–7^ are used to evaluate activities related to daily life. The assessment of ADL alone, including eating, changing clothes, toileting, moving, and bathing, is not sufficient^8,9^ for understanding the life of a TBI patient after reintegration into society. It is also necessary to assess their ability to perform applied activities that are relevant to their daily lives, such as cleaning, washing, shopping, financial management, hobbies, and driving a car. These applied behaviors are influenced by age, sex, education, and lifestyle, and are known in Japan as activities parallel to daily living (APDL).^10,11^

Methods for assessing APDL include the Frenchay Activities Index (FAI).^12–15^ The FAI is an assessment of ADL for individuals living at home devised by Holbrook et al.,^16^ which has been used in stroke patients. The FAI is a 15-item questionnaire-based assessment of applied ADL and social activities, including shopping, meal preparation, outdoor walking, and hobbies. The evaluation method is interview-based, and each item is rated on a scale of 0 to 3 depending on the frequency of the patient’s activities over a 3-month or 6-month period. Although conventional methods of evaluation are based on the status of ADL and the social life of the patient, the FAI is based on the frequency of activities, and can thus provide a better understanding of the patient’s life situation compared with conventional measures. Additionally, the scoring method is simple, and its reliability and validity have been confirmed in Japan as an assessment method for life-related activities. Regarding the FAI evaluation method, Hachisuka et al. reported that the FAI is suitable for the evaluation of stroke patients living at home, because it quantitatively evaluates the performance of applied activities related to life.^11^ Suenaga et al. indicated that the FAI was developed as an evaluation of higher-order functions for stroke patients to live in the community. The credibility and appropriateness of this evaluation method have also been examined, and the FAI is currently considered to be one of the most useful methods of evaluation for stroke patients.

As described above, various studies have investigated APDL among stroke patients after discharge from hospital. However, to the best of our knowledge, no studies have examined this issue in TBI patients. Therefore, the purpose of the current study was to understand the living conditions of TBI patients who have been discharged for more than 1 year after a brain injury using the FAI, which has been used to evaluate the life-related activities of stroke patients, and to clarify their lifestyle characteristics by evaluating the APDL of TBI patients using a questionnaire survey of healthy controls and TBI patients.

## Methods

### Participants

The target group comprised 60 TBI patients (48 men and 12 women) attending a general hospital in Mie prefecture at least 1 year after the occurrence of a brain injury. Additionally, we recruited 788 (326 men and 462 women) healthy participants as a comparison group. The mean age of the TBI patients was 41.4±16.6 years, and the mean time since injury was 1,785±2,134 days (approximately 4 years and 10 months±5 years and 10 months). The healthy participants took part in a community event held in the suburbs of Nagoya, and had a mean age of 44.4±19.6 years. The demographic profiles of the participants are shown in Table 1. Consent to cooperate in the survey was obtained from all healthy participants for this study.

### Survey method

In this study, we used the revised FAI self-assessment chart, which was developed by Holbrook et al. and translated by Shiratsuchi et al.,^17^ and further simplified it in terms of the question options and judgment period (Figure 1). The survey method was an interview-based FAI assessment conducted by an occupational therapist when the TBI patient attended the hospital. For healthy participants, the survey was conducted using an interview format by two occupational therapists and five part-time staff members who received training on the FAI survey at a survey site set up during the hours of a community event. The FAI evaluation consisted of 15 items: shopping, meal preparation, cleaning up after meals, laundry, cleaning and tidying, heavy lifting, going out, walking outdoors, hobbies, transportation use, travel, yard work, house and car maintenance, reading, and work, each rated on a scale of 0 to 3, for a total score of 45 points. The reliability and validity of the revised FAI Self-Assessment Table have been reported by Wade^13^ and Schuling,^15^ and the factor structure has also been examined.^16,18^

The examined items were the total FAI scores, by question item, total scores by sex, age, and occupation. Statistical analysis was performed using the Mann–Whitney U test, and the statistical software was SPSS Statistics 13.0 (IBM).

The study was approved by the ethics review committee of Fujita Health University.

## Results

### FAI total scores and inter-age comparison

The median FAI total score was 22.0 for TBI and 28.0 in healthy participants (Mann–Whitney U test, *P*<0.01). Thus, the TBI patients scored significantly lower than healthy participants (Table 2).

When the FAI total scores were compared by age group, TBI patients were found to have lower scores in all age groups compared with healthy participants (Mann–Whitney U test, *P*<0.05), with significantly lower scores in the 30s, 50s, 60s, and 70s age groups. There was also a trend toward a gradual decrease in scores for TBI patients with age, especially in the 50s and above age groups (Table 3).

### Comparison by questionnaire item

For the FAI questionnaire items, scores for outdoor walking and transportation use were significantly higher among TBI patients than those among healthy participants (Mann–Whitney U test, *P*<0.01). The scores for cleaning up after meals, cleaning and tidying, heavy lifting, shopping, and going out in TBI patients were 2.0 points or more, which were high values (Table 4).

### Comparison of FAI total scores by occupation

Total FAI scores by occupation were significantly lower among TBI patients for unemployed and others (part-time, workshop, etc.) (Mann–Whitney U test, *P*<0.01). On the other hand, healthy participants showed significantly lower values (Mann–Whitney U test, *P*<0.01). For office workers, government employees, high school students, and college students, scores were higher among TBI patients than those in healthy participants, although the differences were not significant (Table 5).

## Discussion

Life-related activities surrounding ADL are known as APDL.^10^ However, there is currently no consensus regarding the concept, scope, and evaluation of these activities.^11,17^ In disease research, several studies have examined applied ADL, such as ADL in stroke patients and instrumental ADL.^19,20^ However, to the best of our knowledge, no studies have examined the evaluation of life-related activities in patients with TBI. Because TBI patients can manifest a wide range of disabilities, including personality changes, emotional disturbances, and difficulty recovering learning behaviors, it would be useful to clarify the status of APDL after discharge, and to determine the associated lifestyle characteristics to consider future therapeutic goals and objectives for TBI patients.

Although the number of TBI patients in this study was relatively small (n=60), most of the patients in this study were in the 30s to 50s age group, in which the rate of traumatic injury from falls and injuries caused by traffic accidents increases. Hence, it is presumed that the activity trend of TBI patients is well reflected. Additionally, we believe that the results of this comparative study are worth considering because the average age of the healthy subjects in the comparison group was in the same range as that of the TBI patients.

Using the FAI, a method for assessing life-related activities, we conducted a comparison of the daily living status of TBI patients more than 1 year after discharge from hospital. This comparison revealed that the level of APDL was significantly reduced in TBI patients in their 50s and older, compared with healthy participants. Although TBI patients in their 20s and 30s have numerous opportunities to attempt to reintegrate into society, such as returning to work or school, the return-to-work rate for middle-aged and older TBI patients is low, and the amount of activity may be reduced because of assistance with household activities by family members who live with the patient.

A variety of mental disorders occur at a high frequency after brain trauma, including somatoform disorders, depressive disorders, and chronic adjustment disorders. In particular, depressive disorders occur in 10% to 77% of brain trauma patients^21^; hence, we expected that patients in our study would not be active, and would tend to remain confined to their homes. Outdoor walking and the use of transportation in the item-by-item comparison of life-related activities indicated that TBI patients were more likely to go outdoors regularly compared with healthy participants. This may have occurred because walking outdoors is a common activity during hospital visits; however, a habit of regular outdoor walking may also be part of rehabilitation. Although the rate of outings to the movies, theater performances, and meetings were significantly lower than those of healthy participants, most TBI patients were found to go out regularly (1–3 times per month). In addition, many TBI patients performed heavy work, such as lifting and lowering blankets, and wiping the floor with a cloth. TBI patients performed shopping at least once a week, at a similar frequency to healthy individuals. Other activities that can be easily undertaken at home by TBI patients included cleaning up after meals, using a broom or vacuum cleaner, and keeping clothes and personal belongings tidy, with similar scores to those of healthy individuals. These activities can be incorporated into rehabilitation for TBI patients regardless of the onset of mental disorder.

A comparison of FAI total scores by occupation between TBI patients and healthy controls revealed that TBI patients scored significantly lower for unemployed and other (part-time, workshop, etc.). Although there were no significant differences, the total FAI scores of TBI patients who were company employees, government employees, high school students, and college students were higher than those of healthy individuals. The results revealed a 10-point difference between TBI patients by occupation, suggesting that TBI patients who belong to organizations, such as companies and schools, tended to work actively toward re-acquiring life-related activities. The fact that there was no difference in the total FAI scores of employees with TBI and healthy individuals suggests that TBI patients may be able to return to work or return to school and regain APDL abilities that were equivalent to those of healthy individuals, although physical and cognitive recovery must be taken into account.

The findings of the current revised FAI Self-Assessment Table demonstrate that TBI patients are less active than healthy individuals but cannot be considered to be inactive. The current results also clarified differences in TBI patients’ engagement in life-related activities and the frequency of activities depending on their social position. Meanwhile, the comparison of TBI patients and healthy participants in this study did not take into account differences in the degree of disability of TBI patients because the TBI patients were not classified according to their level of function. To capture the lifestyle characteristics of TBI patients in more detail, it is necessary to consider the individual abilities of TBI patients, including their level of physical and cognitive functioning.

## Conclusion

Using the FAI to assess life-related activities in stroke patients, we conducted a study to capture the characteristics of the post-discharge lifestyle of TBI patients. Although TBI patients were less active in some life-related activities than healthy participants, they tended to exhibit higher scores for cleaning and tidying, including cleaning up after meals, using a broom or vacuum cleaner, and organizing clothing and personal belongings, with similar scores to healthy participants. This finding indicates that TBI patients should not be considered inactive. The results also clarified differences in TBI patients’ engagement in life-related activities and the frequency of activities depending on their social position. A comparison of FAI items revealed that scores were higher for going outdoors and related activities such as shopping, going out, walking outdoors, and using public transportation, although the differences were not significant. In contrast, scores were significantly lower for yard work, house and car maintenance, and work, suggesting that although TBI patients are not inactive, the majority of their activities are leisure-related rather than work-related. To gain a more detailed understanding of the lifestyle characteristics of TBI patients, it is necessary to take into account the individual abilities of TBI patients, such as their level of physical and cognitive function.

## Figures and Tables

**Table1:** Demographic profiles of enrolled patients

	TBI patients																					Healthy participants																			
	10s			20s			30s			40s			50s			60s			70s			10s			20s			30s			40s			50s			60s			70s	
	Male	Female		Male	Female		Male	Female		Male	Female		Male	Female		Male	Female		Male	Female		Male	Female		Male	Female		Male	Female		Male	Female		Male	Female		Male	Female		Male	Female
Number of participants	3	3		10	2		8	4		11	0		10	2		4	1		2	1		45	76		43	63		46	69		56	55		52	58		39	80		45	61
Age	18.0 (1.0)	12.0 (4.4)		24.6 (2.7)	25.5 (0.7)		34.4 (2.8)	36.3 (2.2)		44.7 (2.5)			54.4 (3.2)	55.0 (0.0)		64.5 (2.7)	66.0 (0.0)		75.0 (2.8)	79.0 (0.0)		16.7 (0.9)	16.8 (1.3)		24.5 (2.7)	23.6 (3.2)		34.4 (2.8)	35.4 (2.5)		43.8 (2.9)	43.4 (2.8)		55.0 (2.7)	55.4 (2.6)		64.1 (2.7)	64.0 (3.0)		73.6 (2.5)	74.2 (3.1)
Spouse																																									
Yes							4	2		4			6	1		3	1		2	1		2	1		9	12		40	68		52	50		49	48		38	70		45	37
No	3	3		10	2		4	2		7			4	1		1						43	75		34	51		6	1		4	5		3	10		1	10		0	24
Co-habitants																																									
Yes	3	3		8	2		7	4		9			9	1		4	1		2	1		44	75		31	50		44	68		55	55		51	56		37	75		45	48
No				2			1			2			1	1								1	2		12	13		2	0		1	0		1	2		2	5		0	13
Occupation																																									
Office worker				2	1		1			3			2									3	1		27	18		35	10		46	6		22	13		6	4		2	0
Civil servant										1												0	0		5	7		6	1		7	3		7	1		1	1		0	0
Housewife																						0	0		0	6		0	37		0	28		0	24		1	50		1	17
Self-employed																						1	0		1	1		3	3		1	1		18	1		9	5		7	1
Farming													1									1	0		1	0		0	0		0	1		1	0		2	2		0	6
Unemployed	1			4	1		5	4		5			6	2		4	1		1	1		0	2		1	5		0	1		0	1		1	9		19	13		24	32
University student	1			1																		4	13		5	17		0	0		0	0		0	0		0	0		0	0
High school student	1	1																				35	54		0	0		0	0		0	0		0	0		0	0		0	0
Other		2		3			2			2			1						1			1	6		3	9		2	17		2	15		3	10		1	5		11	5
Academic background																																									
Elementary school																						0	1		0	0		0	0		0	0		0	0		0	0		6	14
Middle school	2	1		1	1		4			1			2	1		2						40	58		3	1		3	1		0	2		3	13		10	37		17	25
High school	1			7			4	2		4			6	1		2			2	1		5	17		15	33		27	31		23	18		17	38		22	38		11	20
University/Junior college				2				2		4			1									0	0		21	21		15	28		32	29		31	6		5	5		9	1
Other		2			1					2			1				1					0	0		4	8		1	9		1	6		1	1		2	0		2	1

**Figure1:** Revised Frenchay Activities Index

1. Preparing main meals (not including shopping)	
( ) I don’t do this. ( ) I sometimes do this (about 1–3 times per week)	( ) I do this rarely. ( ) I do this more than once per week.

2. Washing up after meals	
( ) I don’t do this. ( ) I sometimes do this (about 1–3 times per week)	( ) I do this rarely. ( ) I do this more than once per week.

3. Washing clothes	
( ) I don’t do this. ( ) I sometimes do this (about 1–3 times per week)	( ) I do this rarely. ( ) I do this more than once per week.

4. Light housework: Cleaning your space with a broom or cleaning device.	
( ) I don’t do this. ( ) I sometimes do this (about 1–3 times per week)	( ) I do this rarely. ( ) I do this more than once per week.

5. Heavy work: Folding up bedding, sweeping floors, moving furniture, or carrying bags.	
( ) I don’t do this. ( ) I sometimes do this (about 1–3 times per week)	( ) I do this rarely. ( ) I do this more than once per week.

6. Local Shopping	
( ) I don’t do this. ( ) I sometimes do this (about 1–3 times per week)	( ) I do this rarely. ( ) I do this more than once per week.

7. Social occasions: movies, theater, eating out, drinking, meeting with friends, etc.	
( ) I don’t do this. ( ) I sometimes do this (about 1–3 times per week)	( ) I do this rarely. ( ) I do this more than once per week.

8. Minutes: walks, shopping, outings longer than 15 minutes.	
( ) I don’t do this. ( ) I sometimes do this (about 1–3 times per week)	( ) I do this rarely. ( ) I do this more than once per week.

9. Actively pursuing q hobby: arts, crafts, sports, other things done for fun (not including watching sports on TV)	
( ) I don’t do this. ( ) I sometimes do this (about 1–3 times per week)	( ) I do this rarely. ( ) I do this more than once per week.

10. Driving a car/going on a bus: using bicycles, cars, buses, trains, airplanes, etc.	
( ) I don’t do this. ( ) I sometimes do this (about 1–3 times per week)	( ) I do this rarely. ( ) I do this more than once per week.

11. Travel on an outing/car ride: Going on recreational trips by car, bus, airplane, etc. (excluding travel for work purposes)	
( ) I don’t do this. ( ) I sometimes do this (about 1–3 times per week)	( ) I do this rarely. ( ) I do this more than once per week.

12. Gardening	
( ) I don’t do this. ( ) I regularly tend to my garden.	( ) I occasionally pull weeds, mow grass, and tend to my garden. ( ) I regularly tend to my garden, and do replanting/potting as needed.

13. Household maintenance	
( ) I do not own these. ( ) I own, and even paint, change designs/upholstery, handle annual inspections, and go for car washes.	( ) I do things like change light bulbs. ( ) In addition to the above, I also do home/car repairs myself.

14. Reading books: Reading habits (not including newspapers, magazines, or pamphlets)	
( ) I do not read. ( ) I read occasionally (approx. once per month.)	( ) I rarely read. ( ) I read often (over twice per month)

15. Gainful work: Full-time, temporary, part-time (any work that provides income, excluding volunteer work)	
( ) I am unemployed. ( ) I work 10–29 hours per week.	( ) I work 1–9 hours per week. ( ) I work over 30 hours per week.

**Table2:** Median total FAI score

	Median	Maximum	Minimum
TBI patients (N=60)	22.0**	35	6
Healthy participants (N=788)	28.0	43	6

** P<0.01 The Mann–Whitney U test was used to compare the differences between TBI patients and healthy participants. Significantly (P<0.01) lower values were marked with **.

**Table3:** Median total FAI scores by age group

	TBI patients (N=60)	Healthy participants (N=788)
10s	19.0 (14.0)	22.0 (7.0)
20s	26.5 (5.5)	28.0 (8.5)
30s	20.5** (9.5)	29.0 (10.0)
40s	28.0 (14.0)	30.0 (11.0)
50s	22.0** (11.0)	30.0 (12.0)
60s	15.0** (10.5)	32.0 (10.0)
70s	12.0* (9.0)	30.0 (9.0)

The interquartile range is shown in parentheses. The Mann–Whitney U test was used to compare the difference between TBI patients and healthy participants. Significantly (P<0.05, P<0.01) low values were marked with * and **, respectively.

**Table4:** Median FAI scores by question items

	TBI patients (N=60)	Healthy participants (N=788)
Meal preparation	0.0**	2.0
Clean-up after meals	2.0*	3.0
Laundry	0.0*	3.0
Cleaning/tidying up	2.5*	3.0
Heavy lifting	3.0	3.0
Grocery shopping	3.0	3.0
Going out	2.0**	3.0
Outdoor walking	3.0	1.0**
Hobby	1.5	2.0
Use of transportation	3.0	2.0**
Travel	0.0**	3.0
Garden work	0.0**	1.0
Home and car care	0.0**	1.0
Reading	0.0**	2.0
Working	0.0**	2.0

* P<0.05 ** P<0.01 Mann–Whitney U test was used to compare the difference between TBI patients and healthy participants. Significantly (P<0.05, P<0.01) low values were marked with * and **, respectively.

**Table5:** Median total FAI score according to occupation

	TBI patients (N=60)	Healthy participants (N=788)
Office worker	29.0 (5.0)	27.0 (11.0)
Civil servant	35.0 (—)	30.0 (9.0)
Self-employed	—	25.0 (10.5)
Housewife	—	33.0 (7.0)
Farming	27.0 (—)	29.0 (11.5)
High school	25.0 (—)	21.0 (7.0)
University	31.5 (—)	25.5** (10.0)
Unemployed	19.0** (12.0)	29.0 (10.5)
Other	22.5** (9.0)	31.0 (8.5)

* P<0.05 ** P<0.01 Interquartile range is shown in parentheses. The Mann–Whitney U test was used to compare the differences between TBI patients and healthy participants. Significantly (P<0.01) lower values were marked with **.
